# Semantic segmentation-based detection algorithm for challenging cryo-electron microscopy RNP samples

**DOI:** 10.3389/fmolb.2024.1473609

**Published:** 2024-10-01

**Authors:** J. Vargas, A. Modrego, H. Canabal, J. Martin-Benito

**Affiliations:** ^1^ Departamento de Óptica, Universidad Complutense de Madrid, Madrid, Spain; ^2^ Department of Macromolecular Structure, National Centre for Biotechnology, Madrid, Spain

**Keywords:** cryo-electron microcopy, semantic segmantation, particle picking, influenza a virus, image proceesing

## Abstract

In this study, we present a novel and robust methodology for the automatic detection of influenza A virus ribonucleoproteins (RNPs) in single-particle cryo-electron microscopy (cryo-EM) images. Utilizing a U-net architecture—a type of convolutional neural network renowned for its efficiency in biomedical image segmentation—our approach is based on a pretraining phase with a dataset annotated through visual inspection. This dataset facilitates the precise identification of filamentous RNPs, including the localization of the filaments and their terminal coordinates. A key feature of our method is the application of semantic segmentation techniques, enabling the automated categorization of micrograph pixels into distinct classifications of particle and background. This deep learning strategy allows to robustly detect these intricate particles, a crucial step in achieving high-resolution reconstructions in cryo-EM studies. To encourage collaborative advancements in the field, we have made our routines, the pretrained U-net model, and the training dataset publicly accessible. The reproducibility and accessibility of these resources aim to facilitate further research and validation in the realm of cryo-EM image analysis.

## Highlights

• Robust methodology for the automatic detection of challenging influenza A virus ribonucleoproteins.
• Outperforms other state-of-the-art cryo-EM particle pickers with practically zero false positives in RNP localization.
• Provides results with near-human accuracy in challenging particle selection tasks.
• Once trained it does not require prior 2D averages or particle data needed and eliminates considerable manual picking workload.

## 1 Introduction

Cryogenic electron microcopy (cryo-EM) single particle analysis is a powerful technique for obtaining high-resolution three-dimensional (3D) reconstructions of macromolecular complexes in a near-to-native state (Merk et al., 2016; Zivanov et al., 2018; Danev et al., 2019). The structural insights obtained from cryo-EM provide a direct way to unravel the mechanisms of the biological reactions driven by these complexes. In the last decade, cryo-EM has undergone a revolution that has pushed it to reach atomic resolution in the determination of structures (Nakane et al., 2020; Yip et al., 2020). This milestone is based on two fundamental pillars: improvements in hardware, mainly in direct electron detectors, and the rapid development of image processing software (Kuhlbrandt, 2014). Now, deep learning algorithms are being integrated into cryo-EM image processing protocols to enhance the capabilities of this technique in structural biology, improving results and simplifying tasks for non-expert users.

High-resolution cryo-EM reconstructions depend on selecting numerous high-quality particles from the micrographs for subsequent image processing. While manual particle picking in micrographs is accurate, it is unfeasible for today’s large datasets due to its time-consuming nature. Consequently, various automatic and semiautomatic methods have been developed. These can be categorized into two types: template-based methods, which rely on reference images for particle selection, and template-free methods that operate without prior information about the particles. Template-free particle picking methods, such as those using Gaussian-generated templates of user-defined size approximating particle dimensions, are noteworthy. Some examples include Relion’s methods (Scheres, 2012), CryoSPARC template picker (Punjani et al., 2017), EMAN2 boxer auto (Tang et al., 2007) or DoG Picker (Voss et al., 2009). These methods facilitate particle selection with minimal prior knowledge and effort. However, they often lack precision in accurately locating particles and may select large amounts of false positives, leading to a preference for template-based methods in high-resolution cryo-EM projects. Template-based methods typically involve manually picking hundreds of particles to obtain 2D reference classes (Tang et al., 2007; Scheres, 2015; Moriya et al., 2017; Punjani et al., 2017; Grant et al., 2018), which are used as patterns for particle selection. Nowadays there is a growing shift towards machine learning/deep learning methods for particle picking, exemplified by tools like XMIPP (Abrishami et al., 2013), SPHIRE-crYOLO (Wagner et al., 2019), EMAN2 (Bell et al., 2018), Topaz (Bepler et al., 2019), APPLE picker (Heimowitz et al., 2018), WARP (Tegunov and Cramer, 2019) or CASSPER (George et al., 2021), among others. These newer methods start with an intensive training phase usually using diverse datasets. This foundational step is designed to train classifiers to recognize cryo-EM particles’ intrinsic features, aiming to enhance accuracy and versatility across different datasets.

The automatic or semiautomatic methods mentioned above have been widely used for boxing both globular macromolecules and mostly straight filament particles. For globular structures, the process involves locating and boxing particle projections to extract them as square subimages, with each containing a full centered macromolecule. Filamentous particles, despite their complex structure, are similarly processed, although the extracted subimages represent only portions of these line-like filaments. However, automatic detection of these particles poses additional challenges compared to globular macromolecules. This is due to their tendency to overlap and intersect, in some cases be curved, and have terminal ends that, from a pattern recognition perspective, differ significantly from the core areas of the filament. It is noteworthy that the study of this type of complexes is crucial as many biologically and medically important proteins are filamentous, making the development of effective automated detection techniques a key focus in structural biology. Prominent examples encompass cytoskeletal proteins such as microtubules and actin, pivotal for various cellular functionalities, including muscle contraction and intracellular cargo transport (Pospich and Raunser, 2018). Moreover, significant instances involve amyloid and tau fibrils, implicated in neurodegenerative pathologies, which have recently garnered heightened attention in structural investigations (Fitzpatrick et al., 2017; Pospich and Raunser, 2017; Scheres et al., 2023). Given the intrinsic difficulty in crystallizing filaments, cryo-EM emerges as the foremost methodology for elucidating their structural attributes.

In previous research efforts, distinct methodologies have been proposed with a primary focus on the identification of linear, filamentous particles (He and Scheres, 2017; Huber et al., 2018; Wagner et al., 2020; Thurber et al., 2021). These approaches leverage the typical inherent characteristics of fibrils, namely their approximate linearity and specific width ranges. To achieve this, various rectangular filters are employed to detect and/or trace filaments, or 2D templates are generated based on previously extracted particles. These methodologies have demonstrated efficacy in the identification and reconstruction of filamentous particles, including but not limited to type 4 filaments (T4F) (Anger et al., 2023), single protofilaments of infectious mouse RML prions (Manka et al., 2022), and structures of tau filaments (Shi et al., 2021). Nevertheless, it is important to note that not all filamentous particles exhibit the characteristic linear conformation. An exemplary case is found in the ribonucleoproteins (RNPs) of the influenza A virus, serving as the epitome of filamentous macromolecular complexes characterized by exceptional flexibility. These RNPs, due to their flexibility and structural diversity, challenge automatic filament pickers and high-resolution reconstruction efforts, with current resolution limitation at ∼7 Å (Coloma et al., 2020). Note that in the 3D reconstructions of these complexes performed to date by our group, the selection of hundreds of thousands of images used was done manually (Arranz et al., 2012; Coloma et al., 2020) as particle picking programs seem not work correctly for this sample. The structural analysis of RNPs and the RNA polymerase in influenza A virus is crucial for understanding the virus infection and proliferation mechanisms. The RNPs of influenza A are complex structures that involve a double helical conformation, playing a key role in mRNA synthesis and genome replication (Arranz et al., 2012). The flexibility and structural heterogeneity of these RNPs, particularly in the context of transcription and replication processes, make them challenging to study but crucial for understanding how the virus replicates and propagates. This understanding can lead to the development of targeted therapies or interventions to manage or prevent influenza epidemics. Importantly, according to the Centers for Disease Control and Prevention in the United States, it is estimated that between 4,900 – 52,000 people died annually due influenza, including influenza A between 2010 and 2022 in the United States with between 100,000 – 710,000 hospitalizations. Thus, understanding the structure and dynamics of RNPs and its RNA polymerase is crucial for comprehending how the influenza virus replicates and transcribes its genetic material, which is a key aspect of its infection mechanism. Nonetheless, the complex details of these processes and the complete understanding of influenza virus infection mechanisms, including all its molecular intricacies, is still not fully understood (Coloma et al., 2020).

In our study, we utilize Semantic Segmentation, a method based on deep learning, to automate the detection of complex Ribonucleoproteins (RNPs) in cryo-electron microscopy images. This includes identifying the locations of RNP filaments and their terminal ends. Importantly, the RNA polymerase, which is crucial for understanding the virus replication, is situated at one end of the RNPs. Therefore, accurately determining its position is vital to determine its structure and thus fully understand the mechanism of virus proliferation. Consequently, our research focused on detecting RNP filaments and their ends. We use a supervised learning approach with a U-net architecture, trained on a small set of manually labeled micrographs. In this process, we label micrograph pixels as either “RNP” or “Background” for RNP filament detection, and “RNP-E” or “Non RNP-E” for RNP ends. Post-training, we have two deep learning models: one for segmenting entire Ribonucleoproteins (Full-RNP model) and another for identifying the ends of the RNPs (RNP-E model). These models enable us to determine the coordinates of both RNPs and their ends. Our results show that this method effectively identifies complex filamentous samples, including RNP filaments and their ends, outperforming other commonly used particle pickers and providing results with near-human accuracy.

## 2 Methods

In this work, we propose two methods to automatically obtain the coordinates of challenging RNP filaments and RNP ends. This section details the raw data used in training and evaluation, along with information on implementation, training specifics, and our processing pipeline.

### 2.1 Biological samples preparation and raw data collection

Our neural networks have been trained and evaluated using as input cryo-EM micrographs of RNPs of the influenza A virus. In the following, we provide details about how this data was produced.

#### 2.1.1 Virus production and RNP purification

The RNPs of the influenza A virus used in this work were produced in the CNB-CSIC from native virions. Virions were purified from cultures of Madin-Darby canine kidney (MDCK) epithelial cells. Cells were infected with Influenza A virus (A/WSN/1933(H1N1)) with a multiplicity of infection of 10^−3^–10^–5^pfu/cell and incubated for 40 h at 37°C. The supernatant was collected when the cytopathic effect reaches 50%. The viruses were isolated using sucrose gradients and centrifugation and lysed to extract the RNPs as described in (Coloma et al. 2009; Coloma et al. 2020).

#### 2.1.2 Sample preparation for electron microscopy

After isolation, RNPs were applied to glow-discharged carbon electron microscopy grids and vitrified by plunge-freezing using liquid ethane. Vitrification is a stain-free, ultrahigh-speed freezing procedure at −180°C that preserves the native structure of the sample and allows the stabilization of unstable complexes or low-life conformers (Arranz et al. 2012; Coloma et al. 2020).

#### 2.1.3 Electron microscopy

The cryo-EM grids were imaged at the ESFR – The European Synchrotron Radiation Facility – in Grenoble, France using a Titan Krios cryo-EM microscope equipped with a K3 direct detector recording 29,493 movies of size 5,760 × 4,092 px with a sampling rate of 0.84 Å/px. Each movie comprising 42 frames and with a defocus ranging from 0.7 to 3 microns. These movies were aligned using MotionCor2 software (Zheng et al. 2017) to correct the drift produce by the electron beam on the sample and the contrast transfer function (CTF) was calculated using GCTF software (Zhang 2016).

#### 2.1.4 Data preparation for the neural network

The aligned micrographs were contrast inverted and downsampled 9 times to produce images of size 640 × 448 px with sampling rate 7.56 Å/px and Fourier Band Pass filtered from 3 to 30 pixels to improve the signal to noise ratio using ImageJ software (Schneider et al. 2012), while other software packages as Relion, Xmipp or EMAN for example could be used as well. These images have enough contrast to visualize the RNP filaments and their ends easily. In the case of the complete RNP filament detection, we manually label 150 of these filtered micrographs, while for the RNP ends detection, we label 500 micrographs.

### 2.2 Neural network architecture and training

For segmenting RNP filaments and RNP filament ends, we implemented a 2D U-net-like convolutional neural network, adapted from Ronneberger et al. (2015). Our neural network architecture comprises three downsampling and three upsampling blocks, each connected with skip connections for feature preservation. Every block includes two convolutional layers activated by RELU functions. The convolutional layers in these blocks use filters of sizes 128, 256, and 512, respectively, each with a kernel size of 7 × 7 to enhance noise robustness. Downsampling in our network is achieved through strided convolutions, while upsampling utilizes transposed convolutions. The final output layer classifies each pixel into two categories, employing a generalized Dice loss function to counteract class imbalance issues. Our network processes images of size 640 × 448 pixels. An essential aspect of our method is the normalization of the input images, where we calculate and apply the 98th and 2nd quantiles for contrast adjustment, clipping values outside the 0–1 range. We divided the labeled dataset into training and validation sets with an 80:20 split. To enhance model robustness, we included random translation transformations (within [−10, 10] pixels range) in the training phase. The model was trained using the Adam optimizer, with batch sizes of 30 images over 50 epochs.

### 2.3 Semantic segmentation processing pipeline

After training the Full-RNP and RNP-E networks, they are applied to segment RNP filaments and their ends across all micrographs. These segmented images are then analyzed to pinpoint their coordinates. Our pipeline for this analysis is as follows:

1. Preprocessing: Each input micrograph is first Fourier *band pass filtered,* downsampled, contrast inverted and normalized as previously described.
2. Model Application: The processed image is fed into either the Full-RNP or RNP-E model to produce a binary segmented image.
3. Post-processing: A closing operation is applied to the binary image to eliminate small gaps. Then, the distinct RNP regions are identified and assigned unique integer labels, based on the connectivity of pixels to their neighbors.
4. Region Filtering: Regions that are too small or too large are automatically excluded.

For processing RNP ends, the centroid coordinates of each labeled region are determined and adjusted by the previously applied downsampling factor. On the other hand, for the processing of RNP filaments, after step 4 the next steps are followed:

5. Skeletonization: For the RNP filaments, the identified regions are skeletonized or thinned using the homotopic thinning algorithm. (Lee et al., 1994), transforming the filament’s thickness into a 1D curve that represents its skeletal structure.
6. Coordinate Calculation: The coordinates of each of these labelled skeletal structures are calculated and adjusted by the downsampling factor previously applied to accurately locate the RNP filament regions.

This pipeline ensures precise and efficient localization of both RNP filaments and their ends in the micrographs.

## 3 Results

In the following, we use the proposed approaches to localize RNP filaments and filament ends in our dataset. We show that our proposed approaches can provide near-human accuracy results and that typical automatic particle pickers do not provide good results in this challenging dataset.

### 3.1 The proposed methods can provide near-human accurate result

In our study, we trained the Full-RNP model utilizing a dataset of 150 micrographs, each meticulously annotated by a human expert. The evaluation of the model’s semantic segmentation predictions, when benchmarked against the ground truth data, provided the outcomes presented in Table 1. To further scrutinize the reliability of manual annotations, a subset of 50 micrographs from the training set underwent dual rounds of manual labeling by the same person, facilitating a comparative analysis of human annotation consistency using identical evaluate metrics. These results, aimed at appraising the precision of human annotations, are shown in Table 1 (a) at row “H-H” and in Table 1 (c).

**TABLE 1:** Evaluation of the model’s RNP-FULL semantic segmentation predictions using conventional metrics for assessing semantic segmentation.

(a)	GAccuracy	MAccuracy	MeanIoU	WeightedIoU	BFSScore
Validation	0.88	0.84	0.70	0.81	0.69
Training	0.88	0.84	0.72	0.80	0.71
H-H	0.86	0.85	0.68	0.78	0.67

(b) (Validation)	Background	RNP	(c) (H-H)	Background	RNP
Background	0.91	0.089	Background	0.87	0.13
RNP	0.24	0.76	RNP	0.18	0.82

(d) (Validation)	IoU	BFSScore	(e) (H-H)	IoU	BFSScore
Background	0.86	0.76	Background	0.84	0.74
RNP	0.58	0.66	RNP	0.51	0.60

(a) Global accuracy (GAccuracy), mean accuracy (MAccuracy), mean intersection over union (MeanIoU), weighted IoU, and the boundary F1 score (BFSScore) metrics calculated for images in the validation and training sets and a subset of 50 micrographs from the training set that underwent dual rounds of manual RNP labeling by the same person (row H-H). (b) Normalized confusion matrix calculated from the validation set. (c) Normalized confusion matrix calculated from the 50 micrographs that underwent dual rounds of manual RNP labeling by the same person. (d) Average per class IoU and BFSScore scores along all images in the validation set. (e) Average per class IoU and BFSScore scores along all images in the image set that underwent dual rounds of manual RNP labeling by the same person.

Table 1 (a) employs a suite of conventional metrics for assessing semantic segmentation, encompassing global accuracy, mean accuracy, mean intersection over union (IoU), weighted IoU, and the boundary F1 (BF) score. Global accuracy (GAccuracy) quantifies the overall proportion of pixels correctly classified across all categories. This metric provides a rapid and computationally efficient assessment of the fraction of pixels correctly classified. Mean accuracy (MAccuracy) calculates the average rate of accurately identified pixels for each category across the dataset. The IoU metric, or Jaccard similarity coefficient, gauges the overlap between the predicted and actual pixels for each class, with MeanIoU averaging this score across all categories. WeightedIoU adjusts the IoU score for each class based on its pixel prevalence, mitigating the influence of minor class discrepancies on the collective metric. The BF score assesses the alignment of predicted class boundaries with their actual counterparts, with MeanBFScore averaging this alignment for each class across all images.

Additionally, Table 1 sections (b) and (c) shows normalized confusion matrices for the background and RNP categories, contrasting the network’s segmentation predictions with the ground truth obtained from the validations set (Table 1 (b)) and juxtaposing the two sets of manual annotations by the same expert (Table 1 (c)). The consistency between the model’s performance on both validation and training sets underscores its robust generalization capability. Finally, Table 1 (d) and (e) shows the intersection over union (IoU) for each class and the average of the BFScore for each class across all images in the validation set and for the subset of 50 micrographs labelled twice for the same person. As can be seen from these results, the congruence of the model’s metrics with those derived from human annotations underscores the model’s potential to achieve near human-level accuracy in RNP semantic segmentation tasks.

**FIGURE 1:**
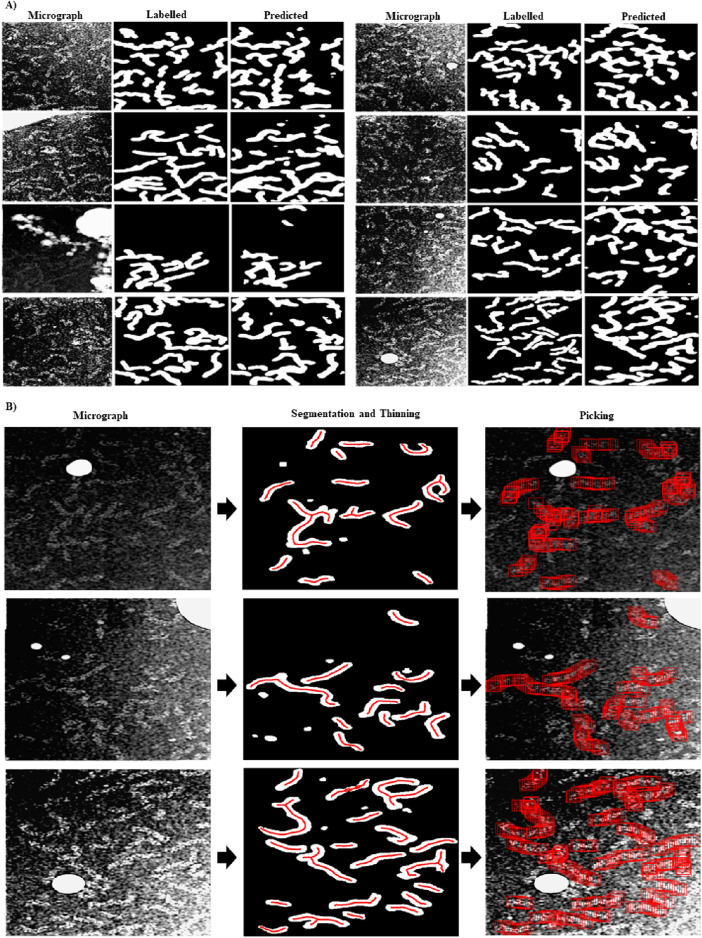
Visual examples showing the performance of the RNP-FULL network with micrographs of the validation set. **(A)** Examples of preprocessed micrographs (micrograph), obtained ground-truth labels by manual labelling (labelled) and predictions made by the RNP-FULL network (predicted). **(B)** In the first, second and third columns, we show respectively preprocessed micrographs, segmented, and filtered images provided by the RNP-FULL network, where the coordinates obtained from the thinning process are shown in red, and the location of the picked particles to be extracted.

In Figure 1A, we show examples of preprocessed micrographs (Fourier *band pass filtered,* downsampled, contrast inverted and normalized), obtained ground-truth labels (labelled) and the predictions made by the RNP-FULL network (predicted). As can be seen from this figure, there is a good visual agreement between the ground truth and the predictions made by the Full-RNP model. In Figure 1B, we show three examples of the processing pipeline followed by our proposed approach. First the preprocessed micrograph is automatically labelled by the trained Full-RNP model, and the segmented images are cleaned, labelled, and thinned. Then, the coordinates of each of these labelled skeletal structures are extracted.

**FIGURE 2:**
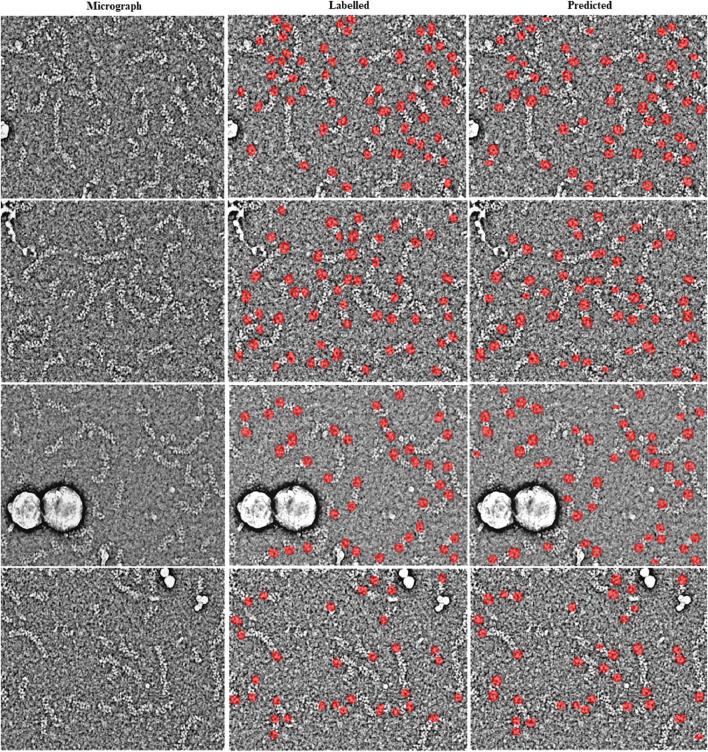
Visual examples showing the performance of the RNP-E network with micrographs of the validation set. Examples of preprocessed micrographs (micrographs), obtained ground-truth labels by manual labelling (labelled) superimposed in red over the corresponding micrograph and predictions made by the RNP-FULL network (predicted) superimposed in red over the corresponding micrograph.

**FIGURE 3:**
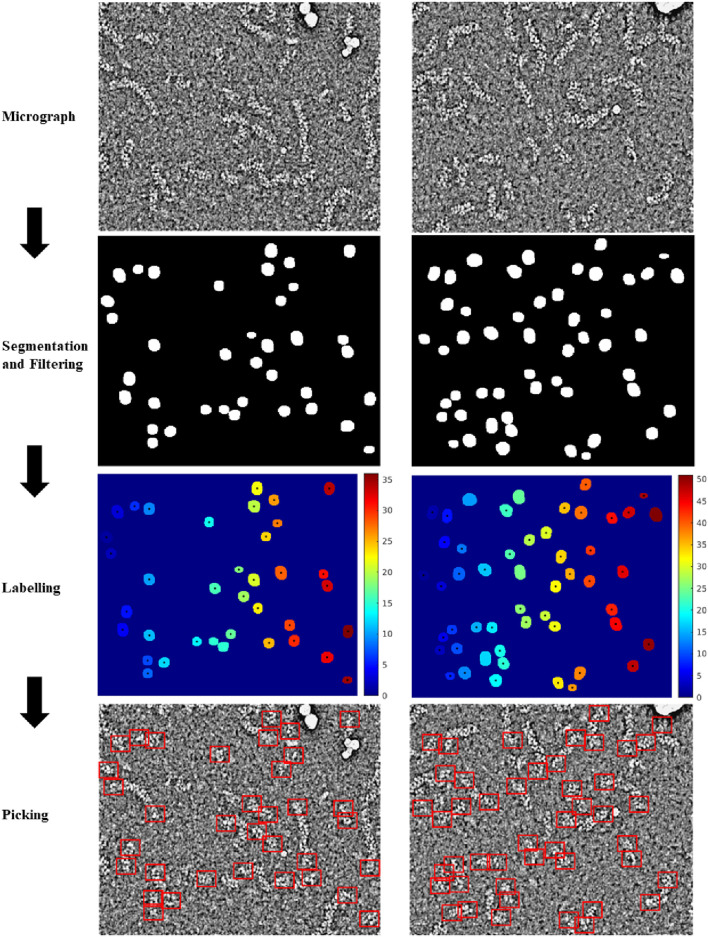
Workflow followed by the proposed method to localize RNP ends. Preprocessed micrographs are segmented automatically by the RNP-E network. These images are labelled with unique integer labels and filtered removing too small and too big regions. For the remaining regions their centroids are computed localizing or picking the ends of the RNPs.

For the training of the RNP-E model, we used 500 manually labelled preprocessed micrographs. In Table 2, we use the same metrics used in Table 1 for assessing semantic segmentation done by the network. In Table 2 (a) row H-H and Table 2 (c) and (e), we show again as reference the results obtained by assessing the accuracy of human annotations for the manual labelling of full RNPs. According to these results, we can concur again the good performance of the RNP-E model and the good similarity between the model metrics and the ones obtained by the same person when labelling the full RNPs showing again the model’s potential to achieve near human-level accuracy in RNP semantic segmentation tasks. In Figure 2, we show examples of preprocessed micrographs (micrographs) and corresponding micrographs with superimposed labelled RNP ends segmented manually (labelled) and predicted by the RNP-E network (predicted). As can be seen from this figure, there is a good agreement between the ground-truth and the automatically segmented RNP ends. Finally, in Figure 3 we show the workflow followed by the proposed method to localize RNP ends. The preprocessed micrographs are segmented automatically by the RNP-E network. Then these images are labelled with unique integer labels based on the connectivity of pixels to their neighbors and too small and too big regions are filtered out. For the remaining regions their centroids are computed localizing or picking the ends of the RNPs.

**TABLE 2:** Evaluation of the model’s RNP-E semantic segmentation predictions using conventional metrics for assessing semantic segmentation.

(a)	GAccuracy	MAccuracy	MeanIoU	WeightedIoU	BFSScore
Validation	0.93	0.82	0.69	0.90	0.73
Training	0.93	0.83	0.69	0.89	0.74
H-H	0.86	0.85	0.68	0.78	0.67

(b) (Validation)	Background	RNP	(c) (H-H)	Background	RNP
Background	0.95	0.05	Background	0.87	0.13
RNP	0.30	0.70	RNP	0.18	0.82

(d) (Validation)	IoU	BFSScore	(e) (H-H)	IoU	BFSScore
Background	0.93	0.79	Background	0.84	0.74
RNP	0.44	0.68	RNP	0.51	0.60

(a) Global accuracy (GAccuracy), mean accuracy (MAccuracy), mean intersection over union (MeanIoU), weighted IoU, and the boundary F1 score (BFSScore) metrics calculated for images in the validation and training sets and a subset of 50 micrographs from the training set that underwent dual rounds of manual RNP labeling by the same person (row H-H). (b) Normalized confusion matrix calculated from the validation set. (c) Normalized confusion matrix calculated from the 50 micrographs that underwent dual rounds of manual RNP labeling by the same person. (d) Average per class IoU and BFSScore scores along all images in the validation set. (e) Average per class IoU and BFSScore scores along all images in the image set that underwent dual rounds of manual RNP labeling by the same person.

### 3.2 Enhanced performance compared to alternative particle pickers

To compare with our deep learning-based picker, we tested the performance of other particle selectors on the same RNP data set. We choose four of the most used pickers: the template matching picker Gautomatch (https://sbgrid.org/software/titles/gautomatch) and the picker included in the Relion software suite (Kimanius et al., 2021) (https://github.com/3dem/relion), Topaz (Bepler et al., 2020) (https://github.com/tbepler/topaz) and CrYOLO (Wagner et al., 2019) (https://pypi.org/project/cryolo/).

**FIGURE 4:**
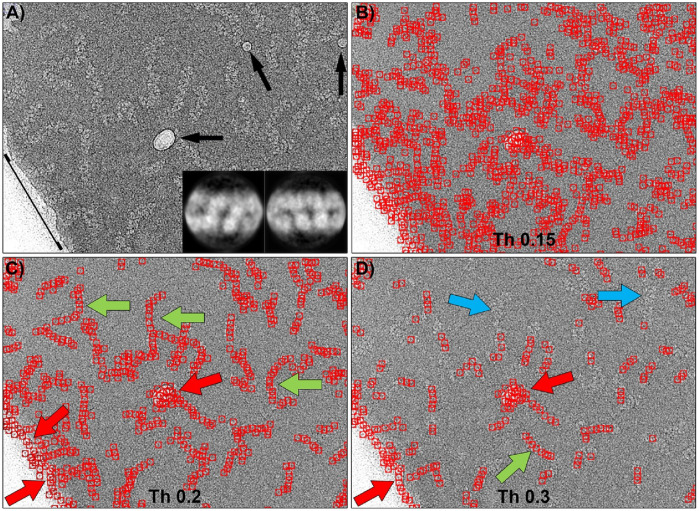
Particle picking of the central region of RNPs using Gautomatch software. **(A)** Typical micrograph showing RNP particles, some ice crystal contamination (black arrows), and the edge of the carbon support layer (black line). The inset shows the 2D averages used as templates for particle selection. **(B)** Particles selected using a threshold of 0.15. At this threshold, there is a large number of false positives distributed throughout the image. **(C)** Particles selected using a threshold of 0.2. Most of the RNP molecules have been correctly picked (green arrows), but ice contaminations and the carbon support edge have also been marked as particles (red arrows). **(D)** Increasing the threshold to 0.3 causes some RNPs to be left undetected by the software (blue arrows), while ice and carbon edge contaminations are still detected as positives.

The template matching algorithm implemented in Gautomatch software requires as main input one or more 2D averages of the particles to be selected, an estimated size of the box that will contain the entire particle, and an estimate of the average minimum distance between two particles in the image. Additionally, there is a tunable threshold value, ranging from 0 to 1, which indicates the level of cross-correlation between the templates and a feature in the micrograph to be considered a positive match. Figure 4 shows the results of Gautomatch picking using 2D averages of the central part of the molecule as templates. The two averages used (inset in panel 4a) were obtained by manually picking and aligning approximately 2000 particles from a random selection of 100 micrographs from the total set of 29,493 images. The panels display the results obtained at different thresholds. Higher values indicate a more restrictive search, where the selected particles are more similar to the 2D averages used as templates. When the threshold is low (0.15), the number of regions selected as positives is very large, including the actual particles and a substantial number of false positives, distributed in the background, in the contaminants, and along the carbon edges of the support. As the threshold value increases (0.2), the RNPs are marked correctly, and the number of false positives selected in the background decreases drastically, although those corresponding to contaminants and carbon edges (red arrows) persist. If the threshold value is increased further in an attempt to reduce the latter false positives (0.3), it is observed that unexpectedly the number of correctly selected particles decreases (blue arrows), while the false positives found in areas of higher contrast remain (red arrows). In summary, there is an optimal threshold (0.2) at which most of the particles are correctly selected, however some contaminations and the edge of the carbon support are also marked as false positives.

**FIGURE 5:**
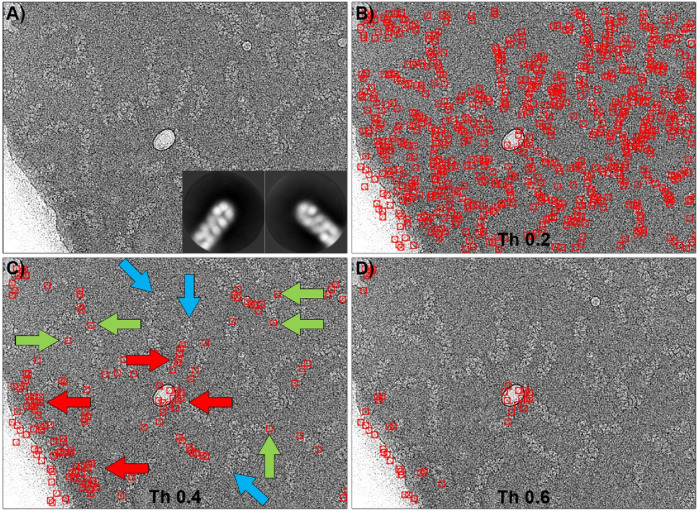
Particle picking of the ends of RNPs using Gautomatch. **(A)** Typical micrograph showing RNP particles. The inset shows the 2D averages used as templates for particle selection. **(B)** Particles selected using a threshold of 0.2. At this threshold, there is a large number of false positives distributed throughout the image, and the picking is very similar to that obtained when the centers of the molecules were used as templates (Figure 4B). **(C)** Particles selected using a threshold of 0.4. Some of the RNP ends have been correctly picked (green arrows), but others have not been detected (blue arrows). Ice contaminations and the carbon support edge have also been marked as positive ends (red arrows). **(D)** Increasing the threshold to 0.6 results in RNPs being undetected, while ice and carbon edge contaminations are still detected as positives.

The ability of Gautomatch to pick the ends of the particles was also tested and the results are shown in Figure 5. Similar to the previous case, the ends of RNPs from 100 micrographs were manually selected and aligned to produce 2D averages, which were used as templates for Gautomatch (inset in Figure 5A). The field covered by the templates in this case was deliberately chosen to be larger than in the previous case to ensure that the image clearly showed the end of the particle, preventing misidentification as an intermediate part of the helix. In this context, multiple tests were performed with different template sizes, and the ones shown here produced the best results. In this case, and very similar to the previous test, using a low threshold (0.2) caused the program to select a large number of matches, including real particles along their entire length (not just the ends) and many false positives. This result is almost indistinguishable from when the 2D averages of the central part of the molecule were used as a template. Increasing the threshold value to 0.4 caused most of the false positives in the background to disappear, and the number of correct positives increased proportionally to the total number of labeled particles (green arrows), although some real ends were no longer selected (blue arrows). However, the number of false positives in the center of the particles and in high-contrast regions (carbon edges and contaminants) remained high (red arrows). Finally, increasing the threshold to 0.6 resulted in the loss of correct positives, with the program selecting only false positives in the high-contrast regions.

**FIGURE 6:**
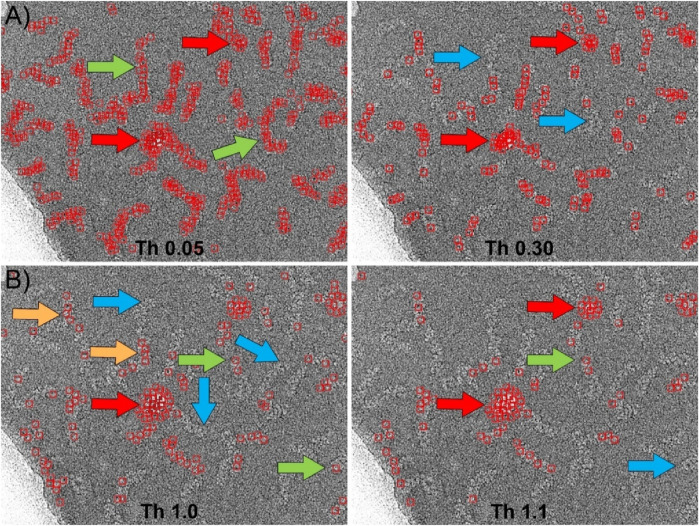
Particle picking using Relion. **(A)** Picking the central region of the RNPs using the 2D averages shown in Figure 4A as templates. After manual optimization of all picking parameters, the most suitable threshold was found to be approximately 0.05. At this threshold, most of the RNPs are correctly selected (green arrows). However, the software also marks areas corresponding to contaminations as positive (red arrows). Increasing the threshold to higher values (Th 0.30) to eliminate these false positives results in the loss of RNPs that were previously correctly marked (blue arrows), while contaminations are still marked as positives (red arrows). **(B)** Picking the ends of the RNPs using the 2D averages shown in Figure 5A as templates. In this case, the detection of the ends was less efficient than for the central regions. Although the software correctly selected a few cases (green arrows), most of the marked positives were actually central regions of the RNPs (orange arrows), leaving many ends unmarked (blue arrows). Contaminations were also marked as positives (red arrows). As in the previous case, increasing the threshold (Th 1.1) caused correctly labeled particles to be lost (blue arrows) while contaminations continued to be detected as positives (red arrows).

We also compared with our particle selector the picking algorithm implemented in Relion (Kimanius et al., 2021). Similar to the previous case, the software requires as main input data the 2D averages to be used as templates, the minimum distance between particles, and two parameters called “minimum mean noise” and “maximum standard deviation noise” designed to prevent the picker from selecting regions of high contrast. The values of these parameters should be determined empirically. Moreover, in Relion there is an adjustable threshold that indicates the level of similarity between the template and the selected feature in the micrograph. Figure 6 shows the results obtained using the same 2D averages from the Gautomatch tests (insets in Figures 4A, 5A) as templates. After empirically optimizing the “minimum mean noise” and “maximum standard deviation noise” parameters to minimize as much as possible the picking of incorrect high-contrast regions, several tests were performed at different thresholds. In the case of the central regions selection (Figure 6A), the results are shown at two thresholds around the optimal value. Using these thresholds, the software correctly identified most of the particles (green arrows), and the edges of the supporting carbon were not marked as positive matches. However, contaminations corresponding to ice crystals were mistakenly selected as particles (red arrows). As with Gautomatch, an increase in the particle selection threshold value resulted in fewer real particles being selected, but the false positives produced by ice crystals remained. Finally, in the particle end-picking test performed using the 2D averages of the insets of Figure 5A as templates, the results were very similar to those obtained using the centers as templates (Figure 6B). Additionally, the selection of regions containing contaminants as false positives (red arrows) also occurred, and increasing the threshold value did not resolve the issue.

In summary, the template-matching-based particle selectors analyzed here produce very similar results whether the central or terminal regions are used as templates, indicating that they can barely discern between these two regions in the images. Although particle selection results are slightly better when using the 2D averages of the central region, the programs tend to select high-contrast regions as positives to some extent, which cannot be resolved by varying the particle selection threshold. However, the results obtained with our software satisfactorily solve these problems and give results similar to those produced by a human expert.

**FIGURE 7:**
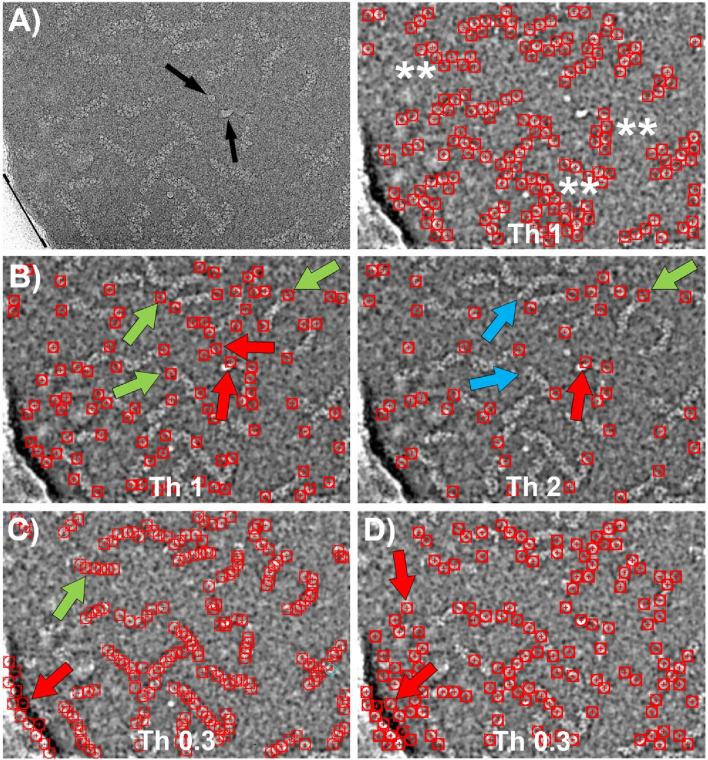
Particle picking using Topaz and CrYOLO software. **(A)** Left: CryoEM image of RNP particles with ice contamination indicated by black arrows and the edge of the carbon support layer marked by a black line. Right: Particles selected by Topaz after training using the central regions of the RNPs with a threshold value of 1. The particles are accurately detected without false positives. However, the coordinate selection occurs near the boundary between the RNP and the background, resulting in a “zigzag” pattern of selected regions (marked with ^**^). **(B)** Particles selected by Topaz after training on the end of the RNPs at two different thresholds. Left: At threshold 1, most of the ends are selected (green arrows). However, there are a small number of false positives due to selection of ice contamination and regions where the RNPs have a sharp bend (red arrows). Right: Increasing the threshold to 2 causes some correct positives that were previously marked to be lost (blue arrows), while some false positives remain (red arrows). **(C)** Particles selected by CrYOLO after training using the central regions of the RNPs. Most of the particles are selected (e.g. green arrow), nevertheless a number of false positives associated to the carbon support are also marked (red arrow). Increasing the threshold does not solve this problem, as correctly picked particles are lost while some false positives remain. **(D)** Particles selected by Cryolo after training using the termini regions of the RNPs. The results obtained are very similar to those shown in **(C)** since the entire particle is selected, rather than just the ends. The number of false positives is higher than in **(C)** (e.g. red arrows).

We also compared the performance of our particle picker against other neural network-based software, specifically Topaz and CrYOLO. The results of these comparisons are presented in Figure 7. To maintain consistency in the evaluation, we used the same set of micrographs and coordinates for training as we did with our own program.

Topaz employs a convolutional neural network based on positive unlabeled learning (Bepler et al., 2020), with multiple adjustable parameters in its learning protocol, including the particle size in its longest dimension. However, for the dataset we used, which includes filamentary and highly flexible structures that often bend, determining an appropriate value for this parameter proved challenging. After extensive trial and error, we found that the renet8 model architecture yielded the best results. Figure 7A shows the particle selection from the central regions of the RNPs at the threshold that produced the optimal outcome. While the particle detection was accurate, with no false positives, the selected coordinates were positioned near the boundary between the particle and the background, rather than at the center of the filaments. This led to the appearance of a “zig-zag” pattern in the coordinates. Figure 7B illustrates the particle selection by Topaz when the network was trained using the ends of the RNPs at two different thresholds. The results were similarly accurate, although there was a slightly higher tendency to select false positives, particularly in areas with ice contamination or sharp bends in the RNPs.

CrYOLO employs a convolutional neural network based on supervised learning, requiring labeled data for training (Wagner et al., 2019). It offers multiple adjustable parameters, such as particle diameter, box size, and detection threshold, to adapt to different datasets. Figures 7C, D display the results of particle picking using CrYOLO, trained on the central regions and the ends of the RNPs, respectively, at the threshold that yielded the best results. Surprisingly, the results are quite similar in both cases, showing little difference between training on centers versus ends. Moreover, when trained on the ends, there was a greater tendency to select false positives.

In summary, neural network-based particle pickers, such as Topaz and CrYOLO, outperform traditional template-matching methods in terms of detection accuracy. However, fine-tuning their parameters is essential and often labor-intensive. Without careful optimization, this can lead to the selection of false positives.

### 3.3 Semantic segmentation picking provides good quality 2D averages

The particle-picking system presented in this study offers not only accurate detection of regions of interest through semantic segmentation, but also introduces a novel method for generating coordinates used for particle extraction. In the case of localizing RNP filaments and unlike other software, which typically calculates the centroid of the detected region to determine coordinate placement, our approach utilizes a skeletonization process. This process identifies the geometric center of the filament, enabling the program to determine the position of the helical axis of the particle, regardless of its curvature. As a result, our software generates a line of coordinates that facilitates the extraction of a continuous series of images along the entire axis of the particle.

**FIGURE 8:**
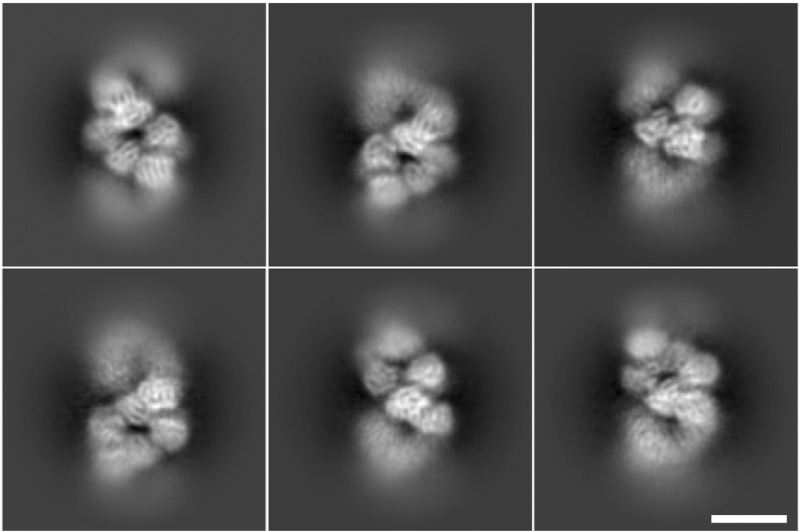
Two-dimensional averages of particles picked using the software presented in this work. All averages are obtained from 800 to 1,000 particles, secondary structure is visible in the nucleoprotein monomer. The scale bar represents 100 Å.

This coordinate generation method for the case of localizing RNP filaments has two significant advantages. First, the extracted images are centered on or near the particle axis, minimizing the shifts required for image alignment. This leads to a reduction in the computational resources needed for image processing. Second, this approach increases the total number of images to be processed in an efficient manner, extending the concept of equispaced and uniform extraction—commonly applied to straight helical particles—to more flexible, curved filaments. Figure 8 shows 2D averages computed with CryoSPARC software (Punjani et al., 2017) of RNP filaments (RNP helical central regions) obtained from particles picked using the software presented in this work, where it is recognizable secondary structure.

## 4 Discussion

As demonstrated by our results, our proposed method performs well and surpasses traditional template-matching pickers. In the localization of complete RNPs, our method has the distinct advantage of having practically zero false positives, whereas template-matching pickers are prone to mistakenly selecting ice contaminations and carbon edges. Surprisingly, increasing the cross-correlation threshold in template matching-based pickers, which theoretically should make the selected particles more closely resemble the templates, tends to result in the selection of incorrect higher-contrast features, picking up false positives representing contaminations and the edges of holes in the carbon. It is important to note that selecting false positives can greatly complicate all subsequent classification and image processing tasks aimed at determining the underlying structure. Compared to other neural network-based pickers, our approach also demonstrates good performance. Neural network-based particle pickers, such as Topaz and CrYOLO, surpass traditional template-matching methods in detection accuracy for localizing RNP filaments and ends. However, these methods require parameter fine-tuning, which can be labor-intensive. Without careful optimization, there is a higher risk of selecting false positives. Our approach seeks to address these challenges, potentially offering improvements in accuracy and efficiency. It is important to highlight that, although the CASSPER method is similarly based on semantic segmentation, it is not well-suited for selecting filamentous particles and their ends. CASSPER is specifically designed for picking globular proteins, as it focuses on estimating the centroids of automatically segmented protein regions.

Furthermore, our method substantially outperforms others in selecting the RNP ends. As previously discussed, the ends of RNPs contain the unique structure of the polymerase, and their study is of great importance, making it crucial to distinguish the ends from other regions of the RNP filaments (RNP helical central regions). In attempts to pick the RNP ends using traditional template-matching pickers, we used 2D averages of images of the RNP ends previously obtained through extensive manual picking followed by 2D particle classification and averaging. Although the templates clearly depicted RNP ends, the results from traditional picker methods were practically the same as those obtained using the central region of the helix as template. Moreover, increasing the threshold to select particles that most resembled the used template led to the same outcome as before, where mainly higher contrast regions were selected that did not correspond to filament ends, thus representing false positives. However, our method correctly selects the filament ends without selecting other filament regions or other false positives (contaminations, carbon edges, etc.), with nearly the same precision as manual picking by a human expert as shown in the result tables. These results suggest that traditional pickers are unable to distinguish between RNP ends and central filament regions, regardless of the template used. This likely occurs because the matching process relies more on the primary structure present in the image (the filament) rather than on the surrounding context, which truly differentiates between central and end regions. In contrast, our method performs exceptionally well in this respect. It is also important to note that our method is capable of selecting features or particles that are sparsely populated in the image (RNP ends), despite their strong resemblance to the majority feature (RNP center filament), with minimal error. This capability is important because it suggests that our picking system could be used to search for minority projections of molecular complexes, which is particularly valuable in structural studies facing the common problem in cryoEM of preferential views in sample preparations.

Our approach has other important advantages. This method does not require as input any prior 2D averages, nor knowledge of any particle data (neither estimated diameter nor minimum distance between particles, etc.). The need for prior knowledge of these parameters complicates the use of other pickers and makes them much more prone to errors if any of those estimates are not precise. Our approach only requires manual segmentation of a limited number of micrographs, overriding the need for parameter knowledge/estimation. Moreover, the typical most reliable way to obtain 2D averages for template matching pickers consist of manual picking on the input micrographs, extract the particles, and align them with existing software. This workflow corresponds to a considerable amount of work. Additionally, alignment software may perform suboptimally when provided with few particles coming from manual picking, leading to poor templates and worse results. All these limitations highlighted before can be overcome when analyzing particles that can be considered “easy to pick”, such as ribosomes, with work and prior experience. However, in challenging cases, these issues can become practically insurmountable, potentially leading to project failure due to poor picking quality. Therefore, although the proposed method has been specifically designed for the localization of the centers and ends of RNPs, we believe that this method holds potential beyond its initial scope. It could be highly beneficial in addressing other complex cases where traditional methods may fall short. Such cases include the picking of very flexible filamentous samples, the selective picking of specific regions within macromolecules, or, as mentioned previously, the picking of minority views that are challenging to identify with conventional techniques. This broader applicability suggests that our method could serve as a valuable tool in a variety of challenging scenarios in the field of image processing and analysis. We believe that in such difficult projects, our approach can significantly facilitate the particle selection task, thereby increasing the probability of success.

## Data Availability

The source code is freely available under the terms of an open-source software license and can be downloaded from https://github.com/1aviervargas/Semantic_Segmentation_Picker. The images used in the training and evaluation of the RNP-E network are available from https://zenodo.org/records/12922653.
